# Mitochondria dysfunction in airway epithelial cells is associated with type 2-low asthma

**DOI:** 10.3389/fgene.2023.1186317

**Published:** 2023-04-21

**Authors:** Lu Zhao, Jiali Gao, Gongqi Chen, Chunli Huang, Weiqiang Kong, Yuchen Feng, Guohua Zhen

**Affiliations:** ^1^Division of Respiratory and Critical Care Medicine, Department of Internal Medicine, Tongji Hospital, Tongji Medical College, Huazhong University of Science and Technology, Wuhan, China; ^2^Key Laboratory of Respiratory Diseases, National Health Commission of People’s Republic of China, Wuhan, China

**Keywords:** T2-low asthma, mitochondria, airway epithelial cells, bioinformatics, hub genes

## Abstract

**Background:** Type 2 (T2)-low asthma can be severe and corticosteroid-resistant. Airway epithelial cells play a pivotal role in the development of asthma, and mitochondria dysfunction is involved in the pathogenesis of asthma. However, the role of epithelial mitochondria dysfunction in T2-low asthma remains unknown.

**Methods:** Differentially expressed genes (DEGs) were identified using gene expression omnibus (GEO) dataset GSE4302, which is originated from airway epithelial brushings from T2-high (n = 22) and T2-low asthma patients (n = 20). Gene set enrichment analysis (GSEA) was implemented to analyze the potential biological pathway involved between T2-low and T2-high asthma. T2-low asthma related genes were identified using weighted gene co-expression network analysis (WGCNA). The mitochondria-related genes (Mito-RGs) were referred to the Molecular Signatures Database (MSigDB). T2-low asthma related mitochondria (T2-low-Mito) DEGs were obtained by intersecting the DEGs, T2-low asthma related genes, and Mito-RGs. Gene ontology (GO) and Kyoto Encyclopedia of Genes and Genomes (KEGG) was performed to further explore the potential function of the T2-low-Mito DEGs. In addition, the hub genes were further identified by protein-protein interaction (PPI), and the expressions of hub genes were verified in another GEO dataset GSE67472 and bronchial brushings from patients recruited at Tongji Hospital.

**Results:** Six hundred and ninety-two DEGs, including 107 downregulated genes and 585 upregulated genes were identified in airway epithelial brushings from T2-high and T2-low asthma patients included in GSE4302 dataset. GSEA showed that mitochondrial ATP synthesis coupled electron transport is involved in T2-low asthma. Nine hundred and four T2-low asthma related genes were identified using WGCNA. Twenty-two T2-low-Mito DEGs were obtained by intersecting the DEGs, T2-low asthma and Mito-RGs. The GO enrichment analysis of the T2-low-Mito DEGs showed significant enrichment of mitochondrial respiratory chain complex assembly, and respiratory electron transport chain. PPI network was constructed using 22 T2-low-Mito DEGs, and five hub genes, *ATP5G1*, *UQCR10*, *NDUFA3*, *TIMM10*, and *NDUFAB1*, were identified. Moreover, the expression of these hub genes was validated in another GEO dataset, and our cohort of asthma patients.

**Conclusion:** This study suggests that mitochondria dysfunction contributes to T2-low asthma.

## 1 Introduction

Asthma is a chronic complicated airway disease characterized by airway hyperresponsiveness, eosinophilic inflammation, mucus hypersecretion, and airway remodeling (Prakash et al., 2017; Durack et al., 2020). More than 300 million people suffer from asthma today and this number is still on the rise (Halayko et al., 2021). The increasing mortality and morbidity rates of asthma leads to the urgent need for new anti-asthma drug development which requires deeper understanding of the molecular mechanisms underlying asthma. Airway inflammation in asthma can be subdivided in T2-high and T2-low subsets (Woodruff et al., 2009; Bhakta et al., 2013; Halayko et al., 2021; Maggi et al., 2022). Clinical biomarkers for T2-high include IgE ≥100 IU/ml, eosinophils count ≥300/μl, and FeNO ≥30 ppb (Busse et al., 2015). In contrast to T2-high asthma, T2-low asthma respond poorly to inhaled corticosteroids (Coverstone et al., 2020). The mechanism underlying T2-low asthma remains largely unknown.

Mitochondria, as fundamental organelles for cellular and systemic metabolism, are critical in the fundamental biological processes including cellular differentiation, apoptosis, autophagy and hypoxic stress responses (AghapourRAPS et al., 2020; Larson-Casey et al., 2020; Zhang et al., 2022). Mitochondria dysfunction has been linked to a variety of disorders including cardiovascular disease, and cancer (Zhang et al., 2021; Vikramdeo et al., 2022; Liu et al., 2023). Moreover, mitochondria play essential roles in various lung diseases. High burden of mitochondrial reactive oxygen species in COPD patients could result in increased mutagenesis (AghapourRAPS et al., 2020). Defects of the mitochondria play an essential role in the apoptosis of airway cells and lung fibrosis (Larson-Casey et al., 2020). Mitochondrial dysfunction has been associated with the pathogenesis of asthma (Chellappan et al., 2022). Airway epithelial cells play a pivotal role in the initiation and development of asthma (Coverstone et al., 2020). However, the role of mitochondria dysfunction in airway epithelial cells in T2-low asthma remains unclear.

Bioinformatics analysis is becoming an important tool in the analysis of the function of genes or proteins in diseases (Dai et al., 2018; Kong et al., 2020; Yang et al., 2020; Wang et al., 2021; Yang et al., 2022). Based on Gene Expression datasets, bioinformatics analysis could potentially reveal the mechanism in asthma. In the present study, bioinformatics analysis using GEO datasets originated from airway brushings were performed and clinical data were verified to investigate the role of epithelial mitochondria dysfunction in T2-low asthma.

## 2 Methods

### 2.1 Data source

The mRNA expression profiles of the airway epithelial brushings were acquired from the GSE4302, GSE67472 datasets. The GSE4302 dataset had 42 asthma subjects and 28 healthy subjects, and Dataset GSE67472 had 62 patients (T2-high: T2-low = 40: 22). According to the Molecular Signatures Database (MSigDB) (http://software.Broadinstitute.org/gsea/msigdb), 1576 mitochondria-related genes (Mito-RGs) were identified (Zhang et al., 2021).

### 2.2 DEGs detection in the airway epithelial brushings from T2-low and T2-high asthma patients

Asthma patients in the GSE4302 dataset were classified as T2-low (n = 20) and T2-high (n = 22) based on the expression levels of signature genes, *SERPINB2, POSTN* and *CLCA1* (Coverstone et al., 2020)*.*

Differential analysis was performed to identify DEGs in T2-low and T2-high patients from the GSE4302 dataset with the empirical Bayes method using the package “limma” R package (4.2.1), with *p* < 0.05 and | log2 (fold change, FC) | > 0.4 as criteria. The volcano map and heat map were also implemented by R package “ggplot2” (version 3.3.6) and “pheatmap” (version 1.0.12), respectively, to graphically display the expression of DEGs.

### 2.3 Gene set enrichment analysis (GSEA)

All genes were ranked according to the degree of expression in T2-low and T2-high asthma patients in the GSEA. Gene Ontology (GO) analysis, in terms of biological processes (BP), molecular function (MF), cell component (CC), was performed to determine if the genes were enriched using the clusterProfiler and R package fgsea (version 1.22.0). According to enrichment scores, top 10 terms were acquired from the above subtypes of GO terms (*p* < 0.05) for the visualized Ridge plots, which were generated using Seurat’s RidgePlot function. Top 5 representative gene sets were shown using the dotplot function.

### 2.4 Identification of T2-low asthma related genes by weighted gene co-expression network analysis (WGCNA)

WGCNA were used to find modules of highly correlated genes. A gene co-expression network was constructed using “WGCNA” R package (version 1.71). Patients was clustered using hclust function to exclude outliers, and the optimal soft-threshold power was set as 6 for further gene clustering. Modules were clustered with more than 300 genes using dynamic tree cut algorithm, and T2-low related modules were recognized using correlation analysis. Relationship between Gene Significance (GS) and Module Membership (MM) was evaluated in the main T2-low related module.

### 2.5 Identification of T2-low-mito DEGs

T2-low-Mito DEGs were identified by intersecting the Mito-RGs, T2-low asthma related genes, and the DEGs obtained from the GSE4302 dataset. The expression of T2-low-Mito DEGs in both T2-low and T2-high groups was analyzed using the Wilcoxon test method. Then, GO and Kyoto Encyclopedia of Genes and Genomes (KEGG) were carried out using R package “clusterProfiler” for signaling pathway analyses of T2-low-Mito DEGs, and the top 10 terms for both GO and KEGG were displayed.

### 2.6 Identification of hub genes

A protein-protein interaction (PPI) network of T2-low-Mito DEGs were constructed in STRING (https://string-db.org/) with a threshold of medium confidence = 0.4. T2-low-Mito DEGs were imported into Cytoscape software (3.9.0) and analyzed by molecular complex detection (MCODE). MCODE was used to identify highly interconnected clusters in a network. Setting the cutoff value as 2, node score as 2, k-score as 2, and max depth as 100, the hub genes were identified in the aimed network cluster. The pROC R package was used for the Receiver operating characteristics (ROC) analyses.

### 2.7 Validation of hub gene levels

The expression of hub genes was confirmed by another dataset GSE67472 with Wilcoxon test method. The box line plots of the hub gene expression in T2-low and T2-high asthma were displayed using Prism 9.0.

### 2.8 Patient recruitment

19 control subjects and 22 asthma patients were collected from Tongji Hospital. Asthma and control subjects were distinguished according to spirometry value and respiratory symptoms. None of the subjects had smoking history or intake of leukotriene antagonist or corticosteroid. For each subject, the demographic information, spirometry as well as fraction of exhaled nitric oxide (FeNO) were measured at the beginning of the study. The diagnosis of asthma and methods for pulmonary function testing and FeNO measurement have been described previously (Wu et al., 2022). The research had been approved by the ethics committee of Tongji Hospital, Huazhong University of Science and Technology.

### 2.9 RNA extraction and real-time quantitative PCR

Total RNA was extracted from human bronchial brushings using TRIzol (Invitrogen, United States), which were further used to generate cDNA with PrimeScript RT reagent kit (Takara, Japan). Primer sequences were designed in Primer-BLAST website (https://www.ncbi.nlm.nih.gov/tools/primer-blast/), and provided by Sangon Biotech, Wuhan, China. The transcript levels were measured on a CFX Connect PCR Platform (Bio-Rad Laboratories, United States) using Takara SYBR Premix ExTaq polymerase. Fold differences were postprocessed using the 2^−ΔΔCT^ method (Wu et al., 2022). The primers used are listed in Table1.

**TABLE 1:** Primers for quantitative PCR.

Symbol	Forward primer sequence (5′-3′)	Reverse primer sequence (5′-3′)
ATP5G1	TTC​CAG​ACC​AGT​GTT​GTC​TCC	GAC​GGG​TTC​CTG​GCA​TAG​C
NDUFAB1	ATG​GCG​TCT​CGT​GTC​CTT​TC	AAC​CTG​CGC​GAG​CAC​TAA​G
TIMM10	TCC​AAG​GGC​GAG​TCT​GTG​T	AAC​TTT​TTG​CCC​ATC​CGC​TCA
UQCR10	ATC​GTG​GGC​GTC​ATG​TTC​TTC	ATG​TGG​TCG​TAG​ATA​GCG​TCC
NDUFA3	GGG​GCC​TCG​CTG​TAA​TTC​TG	GAC​GGG​CAC​TGG​GTA​GTT​G

### 2.10 Statistical analysis

For normally distributed data and non-normally distributed data, we used the means ± standard deviation (SD) and medians (with interquartile ranges) to describe the data, and used unpaired t-test and non-parametric test to compare the groups respectively. All the above were performed using Prism 9.0. *p*-value <0.05 was of statistical significance.

## 3 Results

### 3.1 Identification of the epithelial DEGs between T2-low and T2-high asthma

**FIGURE 1:**
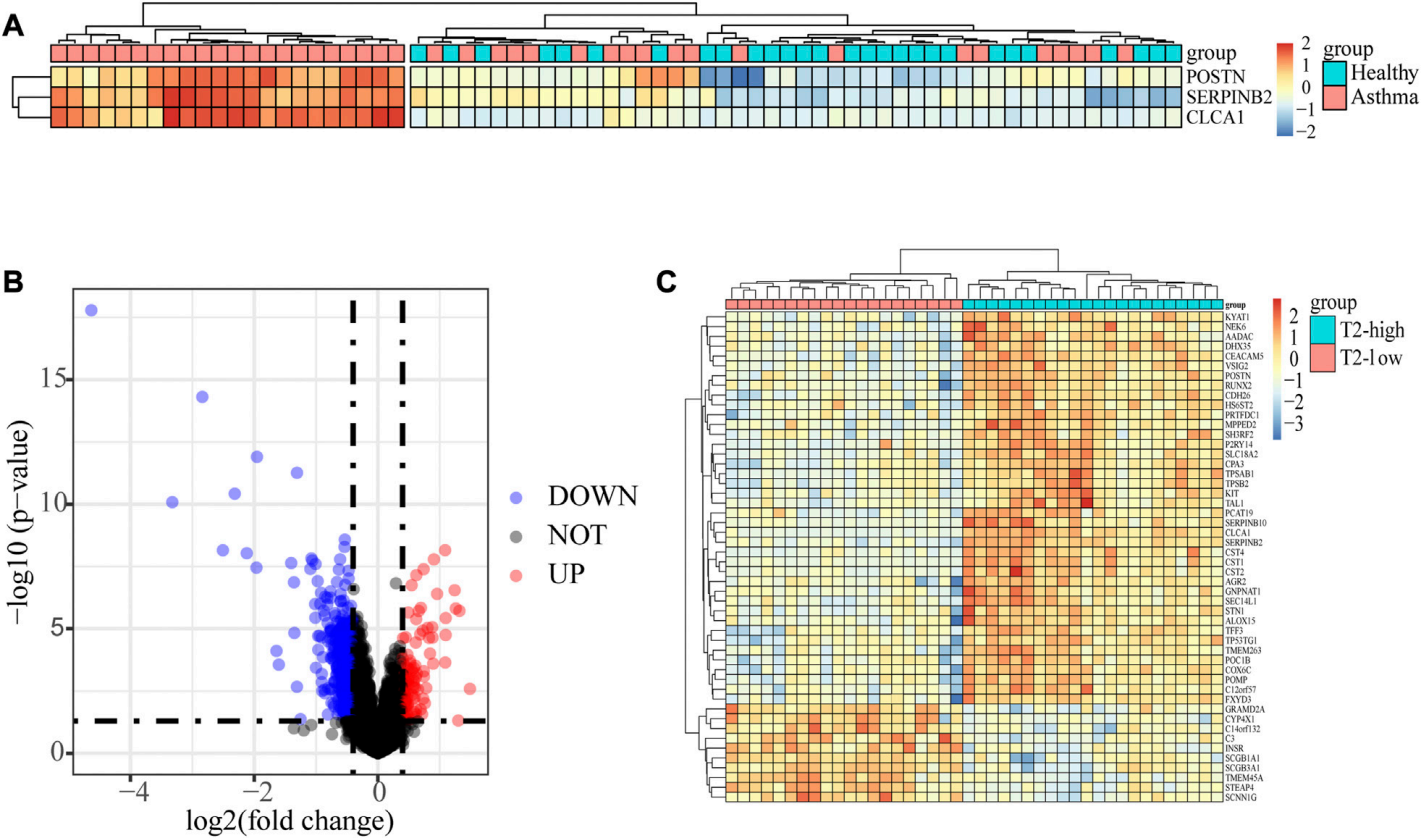
Identification of the epithelial DEGs between T2-low and T2-high asthma. **(A)** Heatmap showing unsupervised hierarchical clustering of POSTN, SERPINB2, and CLCA1 expression levels in bronchial epithelium (red represents a high expression level, and blue represents a low expression level); **(B)** Volcano plot showing DEGs in T2-low vs. T2-high asthma; **(C)** Heat map of Top 50 increased and decreased DEGs (red represents a high expression level, and blue represents a low expression level).

There were 70 subjects including 42 asthma and 28 control subjects in dataset GSE4302. The asthma patients were classified into T2-high asthma (n = 22), and T2-low asthma (n = 20) (Figure 1A) according to the relative expressions of type 2 signature genes, *SERPINB2*, *CLCA1* and *POSTN* (Coverstone et al., 2020). A total of 692 DEGs, including 107 upregulated genes and 585 downregulated genes, were identified between T2-low and T2-high asthma with | log2 FC | > 0.4 and *p* < 0.05. The volcano plot showed upregulated and downregulated and the heat map of the top 50 upregulated and downregulated DEGs results of the DEGs were shown in Figures 1B, C, respectively.

### 3.2 Gene set enrichment analysis (GSEA) of airway epithelial cells from T2-low and T2-high asthma

**FIGURE 2:**
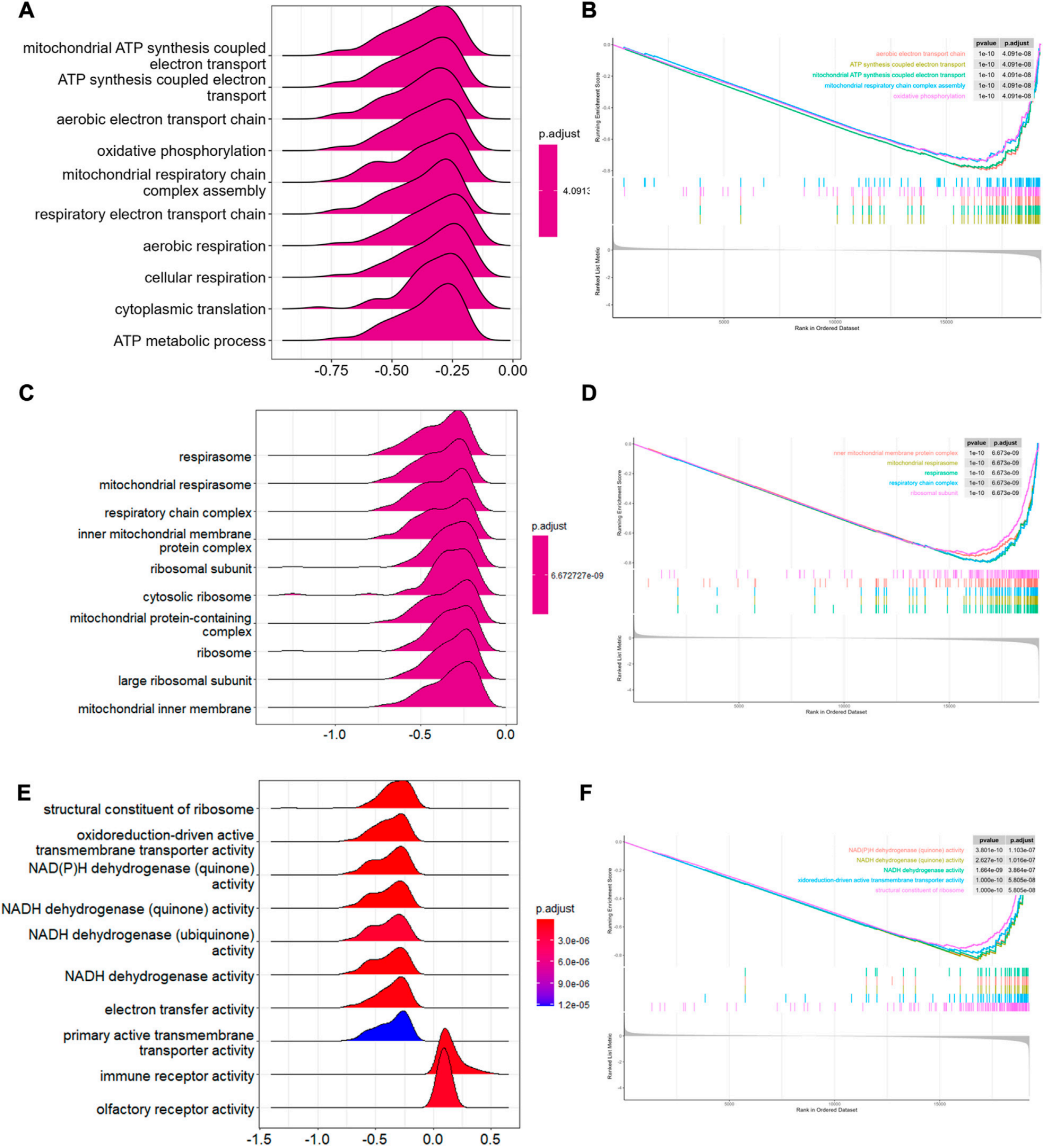
GSEA in T2-low and T2-high asthma. Ridge plots showing GSEA enriched in **(A)** BP, **(C)** CC, **(E)** MF; Top 5 pathways of GSEA enriched in **(B)** BP, **(D)** CC, **(F)** MF.

To analyze the potential biological pathway involved in T2-low and T2-high asthma in GSE4302, GSEA was performed using GO term enrichment with BP, MF, and CC. With the implementation of ridgeplots, the most significantly biased ridges in each ontology were demonstrated according to the adjusted *p*-value. Top 10 up- and downregulated GO terms are shown in Figures 2A, C ,E. The top 5 were further showed with gseaplot2 in Figures 2B, D, F. We found that the top 3/10 GO terms in BP ontology including: mitochondrial ATP synthesis coupled electron transport the mitochondrial, mitochondrial electron transport NADH to ubiquinone, and mitochondrial respiratory chain complex assembly. Of note, the top 4/10 GO terms in CC ontology including mitochondrial respirasome, inner mitochondrial membrane protein complex, mitochondrial protein-containing complex and mitochondrial inner membrane, are also significantly associated with T2-low asthma. Likewise, NADH dehydrogenase and oxidoreductase involved in regulating the MF progression related to mitochondrial respiratory electron chain complex. These data suggests that mitochondria related pathway play a key role in T2-low asthma.

### 3.3 Identification of T2-low asthma related genes by weighted gene co-expression network analysis (WGCNA)

**FIGURE 3:**
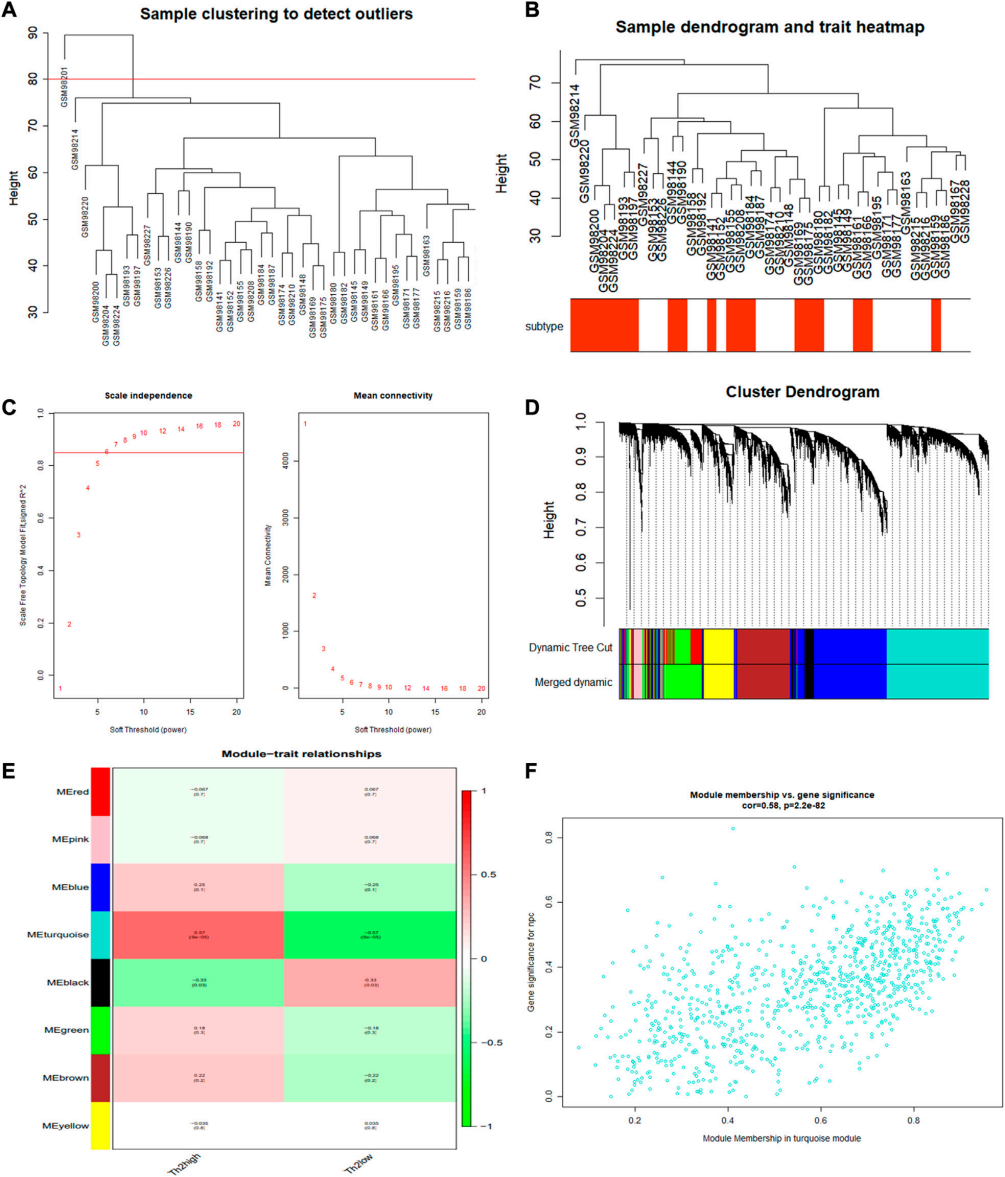
WGCNA performed for identifying gene modules significantly related to T2-low asthma. **(A)** Sample clustering; **(B)** Heat map of sample clustering and its characteristics; **(C)** Filtering of soft thresholds; **(D)** Clustering dendrogram of differentially expressed genes; **(E)** Heatmap of the correlation between module and clinical traits (each cell contained the correlation coefficient and corresponding *p*-value); **(F)** The gene significance for T2-low asthma in the turquoise module (one dot represents one gene in the turquoise module).

One outlier sample (GSM98201) was excluded from the GSE4302 dataset using the hierarchical agglomerative clustering method with the cutoff value as 80 (Figure 3A). The remaining 41 cases were clustered according to their relative Euclidean distance for further gene analyses (Figure 3B). Performing WGCNA, and selecting the scale-free fitting index as 0.85, we got the optimal soft threshold power value, which was 6 and led to the mean connectivity being around 100 (Figure 3C). Eight modules were obtained using the dynamic shear tree algorithm with the minimum 300 genes (Figure 3D). Module-trait relationship analyses showed that the turquoise module strongly correlated with T2-low Asthma, and the 904 genes in the turquoise module were regarded as T2-low Asthma related genes (Figure 3E). The genes in the turquoise module showed high correlation between MM and GS (Figure 3F).

### 3.4 Identification of T2-low-mito DEGs

**FIGURE 4:**
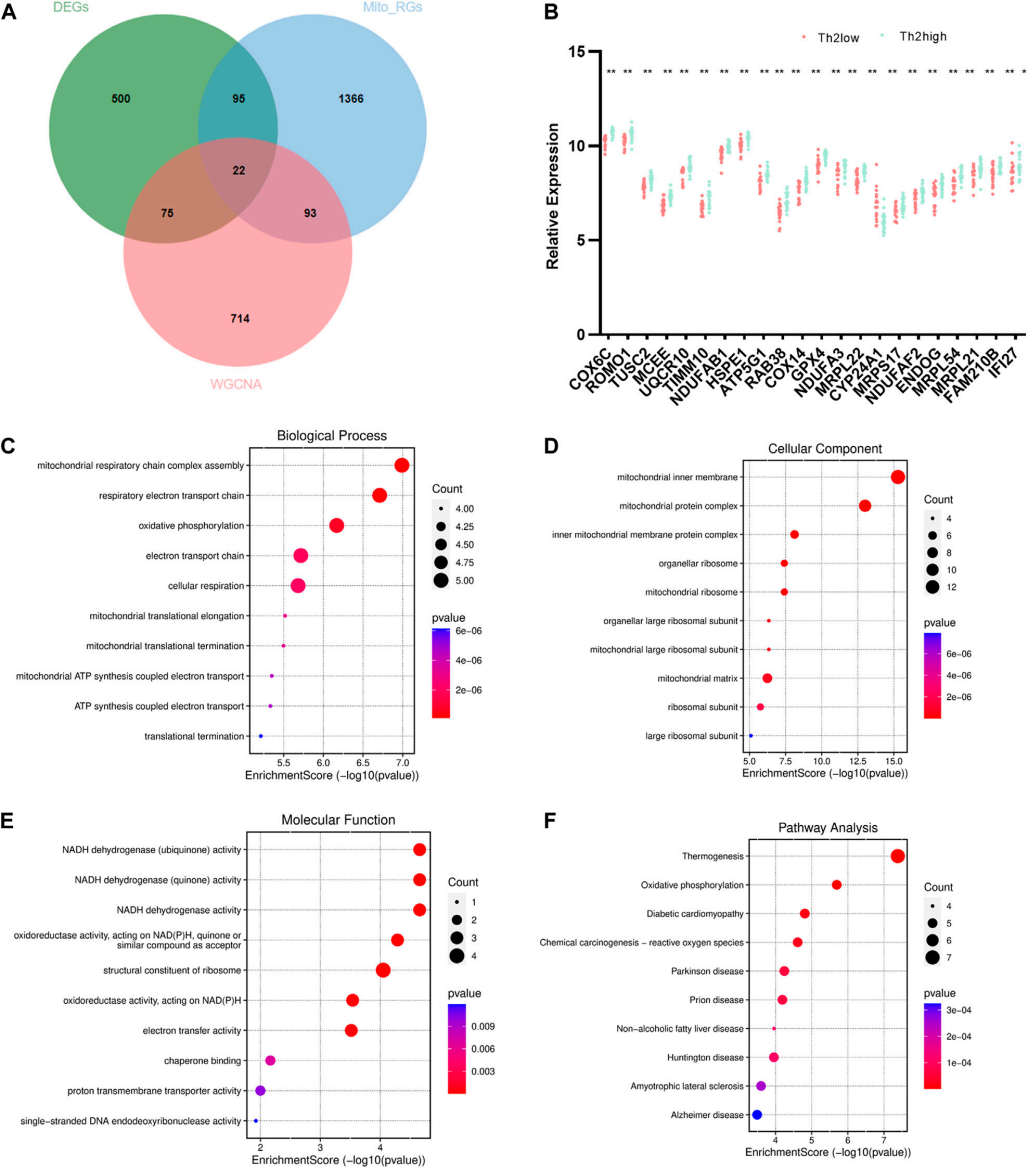
Identification and enrichment analysis of T2-low-Mito DEGs. **(A)** Venn diagram of DEGs, turquoise module, and Mito-RGs; **(B)** Dot plot of 22 T2-low-Mito DEGs in T2-low vs. T2-high asthma; Go enrichment results (Top 10) enriched in **(C)** BP, **(D)** CC, **(E)** MF; **(F)** KEGG enrichment results (Top 10). *p < 0.05, **p < 0.01.

A total of 22 T2-low-Mito DEGs were identified by intersection, as shown in the Venn diagram (Figure 4A). A comparison analysis revealed that *COX6C, ROMO1, TUSC2, MCEE, UQCR10, TIMM10, NDUFAB1, HSPE1, ATP5G1, RAB38, COX14, GPX4, NDUFA3, MRPL22, CYP24A1, MRPS17, NDUFAF2, ENDOG, MRPL54, MR21, FAM210B,* and *IFI27* were significantly less expressed in T2-low asthma than in T2-high asthma (Figure 4B). The top 10 enriched signaling pathways in both GO and KEGG analyses were shown in Figures 4C–F. Mitochondrial respiratory chain complex assembly, and respiratory electron transport chain were enriched prominently in GO analyses. Similarly, the KEGG pathway enrichment also covered thermogenesis and oxidative phosphorylation.

### 3.5 Identification of hub genes

**FIGURE 5:**
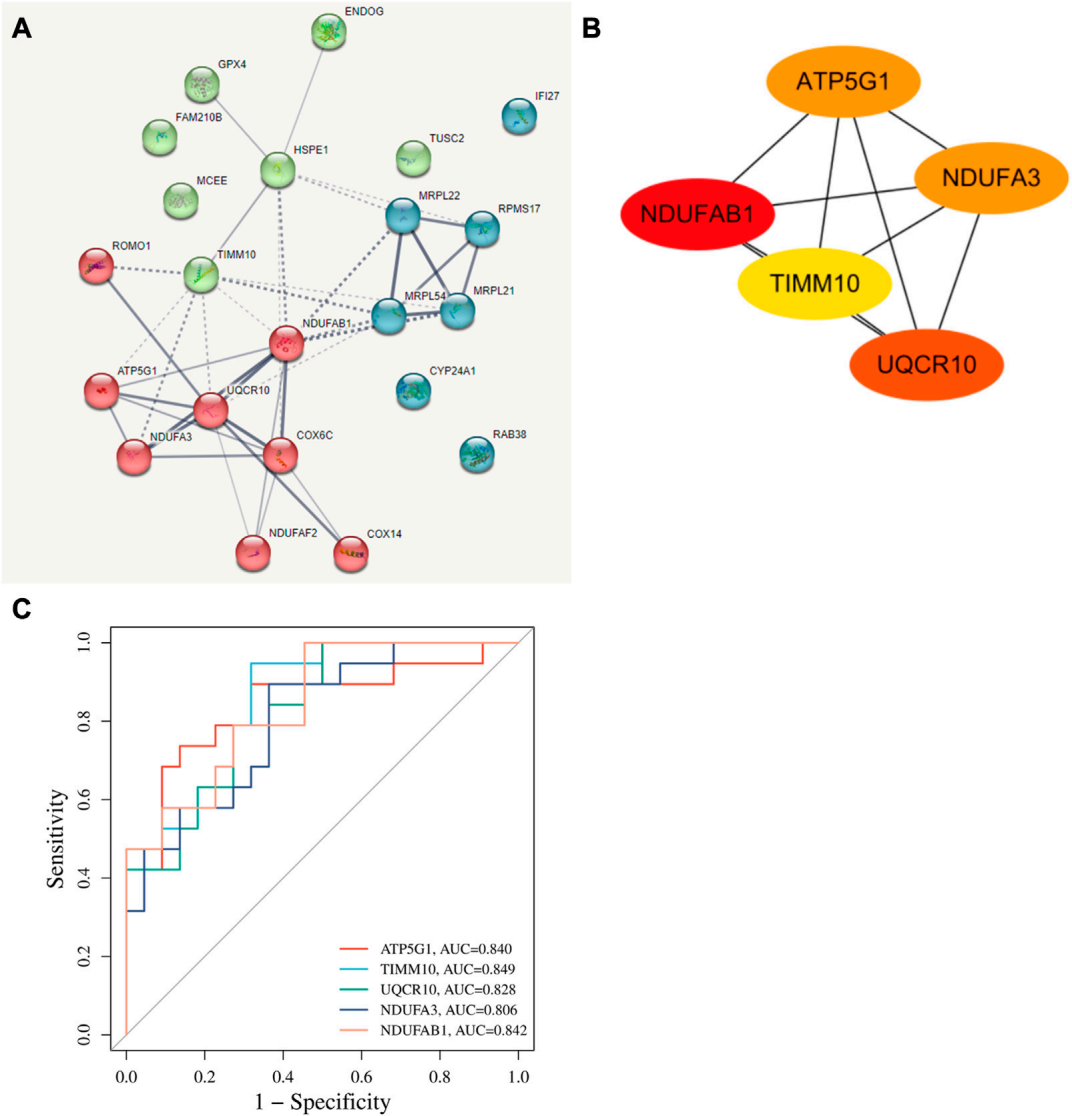
Identification of hub genes. **(A)** PPI network predicting highly potential interactions with 22 T2-low-Mito DEGs based on SRTING database; **(B)** Identification of hub genes from the PPI network with Cytoscape plug-in MCODE; **(C)** ROC curve of hub genes in dataset GSE4302.

The interaction of 22 T2-low-Mito DEGs were predicted by PPI network using the STRING website (Figure 5A). We next performed subnetwork analysis of the PPI network to identify significant cluster enriched in T2-low-Mito DEGs by MCODE cluster analysis in Cytoscape. Of note, all 22 DEGs in PPI network were used to select hub genes, and five hub genes were identified, including *ATP5G1, UQCR10, NDUFA3, TIMM10,* and *NDUFAB*1 (Figure 5B). In addition, the significant diagnostic value for T2-low asthma of these hub genes in dataset GSE4302 were validated by the ROC curve (Figure 5C).

### 3.6 Validation of the hub genes

**FIGURE 6:**
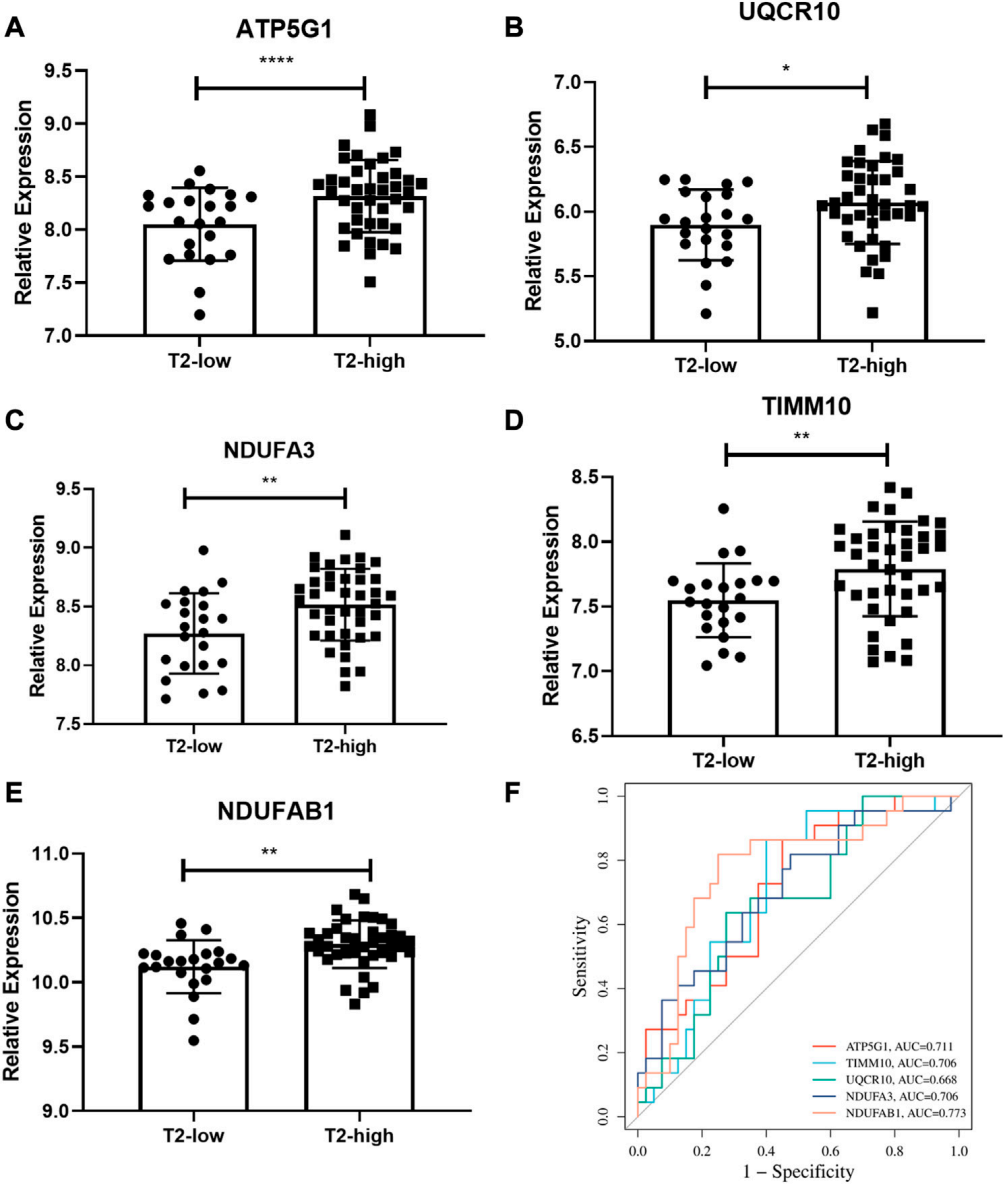
Validation of the hub gene levels. **(A–E)** GSE67472 data set was used to verify the hub gene levels of *ATP5G1, UQCR10, NDUFA3, TIMM10, and NDUFAB1*; **(F)** ROC curve of hub genes in dataset GSE67472. *p < 0.05, **p < 0.01, ****p < 0.0001.

The expressions of the five hub genes, *ATP5G1, UQCR10, NDUFA3, TIMM10,* and *NDUFAB*1, between the T2-low and T2-high group were validated in another dataset GSE67472, as shown in Figure 6. These genes were also poorly expressed in the T2-low group with Wilcoxon test, consistent with the results of GSE4302.

**FIGURE 7:**
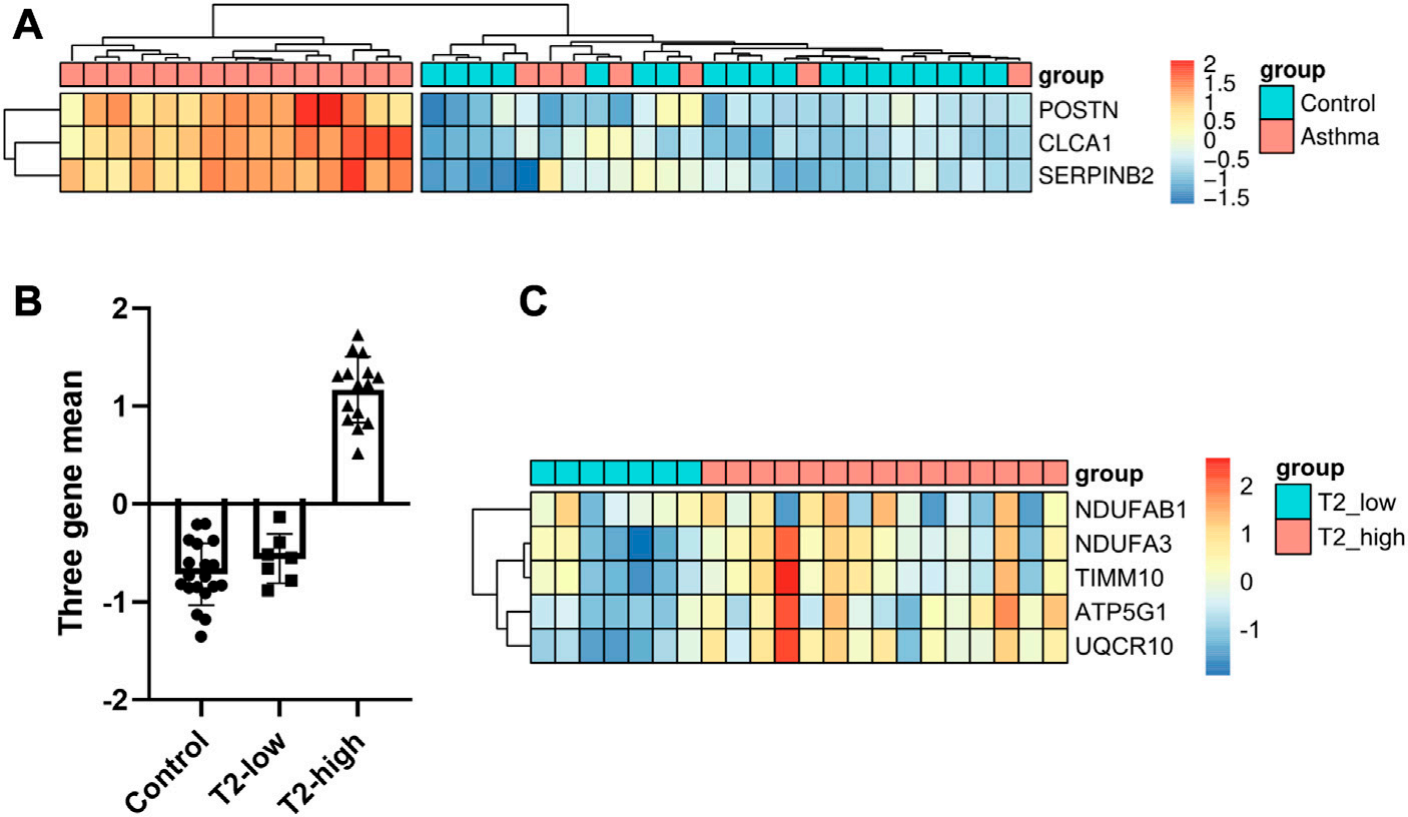
Identification of DEGs between T2-low and T2-high asthma in clinical samples. **(A)** Heatmap showing unsupervised hierarchical clustering of POSTN, SERPINB2, and CLCA1 expression levels in bronchial epithelium (red represents a high expression level, and blue represents a low expression level); **(B)** Dot plot of three-gene mean of POSTN, SERPINB2, and CLCA1 expression levels in control, T2-low and T2-high asthma; **(C)** Heat map of the five hub gene levels (red represents a high expression level, and blue represents a low expression level).

**FIGURE 8:**
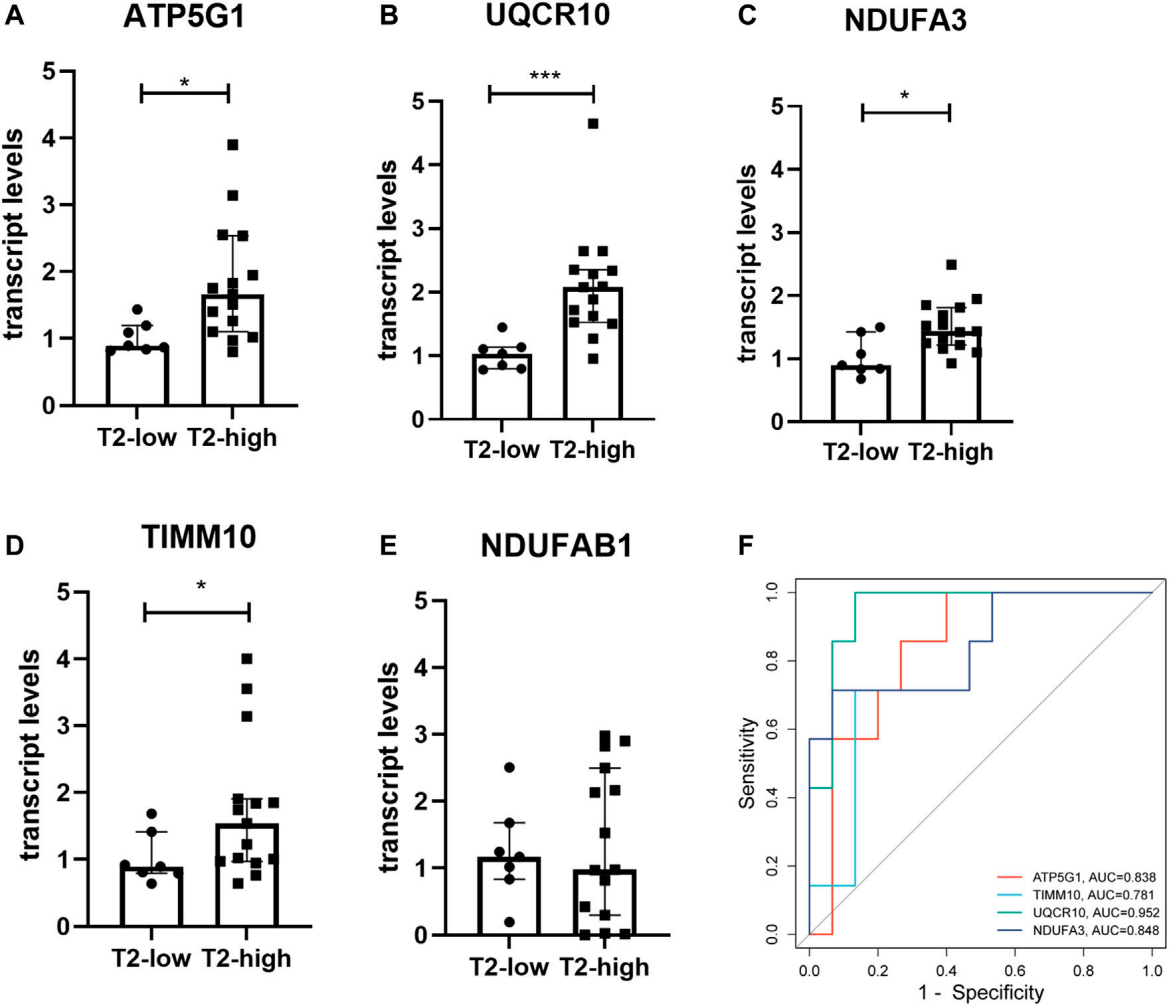
Validation of the hub gene levels in clinical samples. **(A–E)** qRT-PCR was used to verify the hub gene levels of *ATP5G1, NDUFAB1, UQCR10, NDUFA3, and TIMM10* in bronchial brushings from T2-high asthma patients (n = 15) and T2-low asthma patients (n = 7). *p < 0.05, ***p < 0.001. **(F)** ROC curve of hub genes in clinical samples.

We recruited 19 healthy control subjects and 22 asthma patients to further validate the expression of the hub genes. Subject characteristics of the healthy control subjects and asthma patients are summarized in Table 2. Furthermore, we classified the asthma patients into T2-high asthma (n = 15), and T2-low asthma (n = 7) (Figures 7A, B). The characteristics in the two subsets of asthma patients are summarized in Table 3. The expression levels of the five hub genes were determined by qRT-PCR and the expression levels were relative to T2-low asthma, which was shown in heatmap (Figure 7C) and bar chart (Figures 8A–E). The experimental results of *ATP5G1, UQCR10, NDUFA3,* and *TIMM10* were consistent with the bioinformatics analysis results with highly diagnostic value (Figure 8F).

**TABLE 2:** Overall subject characteristics.

	Healthy control subjects	Subjects with asthma	*p*-value
Number	19	22	
Age, y	40.58 ±11.02	45.95 ±12.94	0.1638
Sex, M: F, %F	7:12 (63.16)	8:14 (63.64)	0.9999
Body mass index	21.57 ±2.741	22.26 ±2.332	0.3854
FEV_1_, %predicted	97.40 (73.90–115.2)	80.25 (34.20–97.50)	<0.0001
FeNO, ppb	14 (3.7–38); n = 15	67.50 (9–196); n = 22	<0.0001

Data are shown as frequencies (percentages), means ± SDs, or medians (ranges).

FEV_1_, Forced expiratory volume in the first second; FeNO, Fraction of exhaled nitric oxide.

**TABLE 3:** Subject characteristics and bronchoscopic features by asthma phenotype

	T2-low	T2-high	*p*-value
Number	7	15	
Age, y	47.43 ±10.20	45.27 ±14.33	0.7247
Sex, M: F, %F	2:5 (71.43)	7:8 (53.33)	0.6478
Body mass index	20.91 ±2.355	22.33 ±2.207	0.1839
FEV_1_, %predicted	81.89 ±7.120	75.57 ±17.98	0.2530
FeNO, ppb	22.63 ±18.22	104.2 ±52.31	<0.0001

Data are shown as frequencies (percentages), means ± SDs.

FEV1, Forced expiratory volume in the first second; FeNO, Fraction of exhaled nitric oxide.

## 4 Discussion

Asthma is a chronic complicated airway disease, and presents a high prevalence throughout the world (Naumova et al., 2022). Airway inflammation in asthma can be subdivided in T2-high and T2-low subsets based on molecular mechanism (Halayko et al., 2021; Maggi et al., 2022). T2-low asthma can be severe and corticosteroid resistant, and targeted interventions for T2-low asthma have been mostly unsuccessful (Durack et al., 2020). The understanding of the mechanism underlying T2-low asthma is relatively limited.

Severe asthma is associated with energy metabolism dysfunction (Xu et al., 2022), and mitochondria play an important role in energy metabolism (Zhang et al., 2022). Bronchial epithelial cells play an essential role in asthma and mitochondrial dysfunction is implicated in asthmatic bronchial epithelial cells (AghapourRAPS et al., 2020). Drugs that improve mitochondrial dysfunction can be used for the treatment of asthma (Chellappan et al., 2022). However, the role of epithelial mitochondrial dysfunction in T2-low asthma remains unknown.

Our analysis showed that mitochondrial ATP synthesis coupled electron transport the mitochondrial is involved in T2-low asthma by analyzing all the gene expression. GSEA revealed that the significantly enriched pathways focusing on mitochondrial dysfunction. This suggests that mitochondrial dysfunction plays an important role in T2-low asthma. To further investigate the core mechanism and hub genes, T2-low asthma related genes were obtained by WGCNA, and gene networks were constructed to find the potential pathways underlying T2-low asthma.

Furthermore, GO enrichment analysis on T2-low-Mito DEGs showed that mitochondrial dysfunction was strongly related to T2-low asthma. T2-low-Mito DEGs mainly focused on mitochondrial inner membrane, mitochondrial ribosome, and mitochondrial matrix in Cell Component, which cooperate with each other to exert NADH dehydrogenase activity, oxidoreductase activity, and electron transfer activity in Molecular Function, . In the category of Biological Processes, T2-low-Mito DEGs were mainly focused on mitochondrial respiratory chain complex assembly, respiratory electron transport chain, oxidative phosphorylation, electron transport chain. The mitochondrial respiratory chain, also known as the electron transport chain, lies in the inner mitochondrial membrane where oxidative phosphorylation takes place (Zhang et al., 2022). The oxidative phosphorylation system (OXPHOS) consists of multiple respiratory chain complexes (I-V) (Vikramdeo et al., 2022). Besides, superoxide and other reactive oxygen species (ROS) generated by the mitochondrial respiratory chain during oxidative phosphorylation, play an essential role in asthma (Sharma et al., 2021).

We identified five hub genes (*ATP5G1, UQCR10, NDUFA3, TIMM10,* and *NDUFAB1*) from T2-low-Mito DEGs using MCODE in the Cytoscape. *ATP5G1*, a key element of OXPHOS, encodes mitochondrial ATP synthase and promotes ATP synthesis (Sun et al., 2021). During ATP synthesis through oxidative phosphorylation in mitochondria, decreased *ATP5G1* lead to that the mitochondrial respiratory chain created a lower electrochemical gradient and generates mitochondrial membrane potential (MMP) declined. Decreased MMP indicates mitochondrial dysfunction and apoptosis (Prakash et al., 2017). In clear cell renal cell carcinoma, and the deficiency of *ATP5G1* contribute to mitochondrial disorders (Brüggemann et al., 2017). Therefore, we speculate that decreased MMP may be the mechanism of T2-low asthma.

*UQCR10*, a subunit of complex III of the electron transport chain, takes part in the oxidative phosphorylation of the inner mitochondrial membrane (Páleníková et al., 2021). Recently, the function of *NDUFA3* in mitochondrial electron transport chain complex I gains academic attention (Rak and Rustin, 2014). One study has demonstrated *NDUFA3* are downregulated in Clear-Cell Renal-Cell Carcinoma (Brüggemann et al., 2017). However, the existing evidence on the role of *NDUFA3* in asthma is insufficient, so further investigations are needed. Taken together, downregulation of *ATP5G1, UQCR10* and *NDUFA3* contribute to the decreased ATP production, while decreased ATP production was accompanied with the increase of mitochondrial ROS generation. This indicates that dysregulation of ATP and ROS production contributes to the pathogenesis of T2-low asthma.

*TIMM10* is implicated in the entry of essential proteins into inner membranes of mitochondria (Ma et al., 2013). Another hub gene identified in our study, *NDUFAB1*, contributes to mitochondrial activities and ROS metabolism through regulating the complexes of the electron transport chain. However, the role of *TIMM10* and *NDUFAB1* in asthma requires further investigation.

There are several limitations of our study. First, the sample size of the datasets from GEO and our own cohort are relatively small. Second, although we validated the aberrant expression of the hub genes in another dataset and our cohort of asthma patients, the role of the hub genes in T2-low asthma requires further study.

## 5 Conclusion

In this study, we identified aberrant mitochondrial pathways in T2-low asthma and five novel hub genes related to both of T2-low asthma and mitochondria dysfunction. This suggests that mitochondria dysfunction contributes to T2-low asthma, providing new clues for the diagnosis and therapy for T2-low asthma.

## Data Availability

The original contributions presented in the study are included in the article/supplementary material, further inquiries can be directed to the corresponding authors.
